# Central poststroke pain: somatosensory abnormalities and the presence of associated myofascial pain syndrome

**DOI:** 10.1186/1471-2377-12-89

**Published:** 2012-09-11

**Authors:** Rogério Adas Ayres de Oliveira, Daniel Ciampi de Andrade, André Guelman Gomes Machado, Manoel Jacobsen Teixeira

**Affiliations:** 1 Pain Center, Department of Neurology, School of Medicine, University of São Paulo, São Paulo, Brazil; 2 Pain Center, Instituto do Câncer do Estado de São Paulo, São Paulo, Brazil; 3 Functional Neurosurgery Center for Neurological Restoration, The Cleveland Clinic Foundation, Cleveland, USA; 4 Divisão de Clínica Neurológica do Hospital das Clínicas da FMUSP Secretaria da Neurologia, Instituto Central, Av. Dr. Enéas de Carvalho Aguiar, 255, 5° andar, sala 5084 – Cerqueira César, São Paulo, SP, 05403-900, Brazil

## Abstract

**Background:**

Central post-stroke pain (CPSP) is a neuropathic pain syndrome associated with somatosensory abnormalities due to central nervous system lesion following a cerebrovascular insult. Post-stroke pain (PSP) refers to a broader range of clinical conditions leading to pain after stroke, but not restricted to CPSP, including other types of pain such as myofascial pain syndrome (MPS), painful shoulder, lumbar and dorsal pain, complex regional pain syndrome, and spasticity-related pain. Despite its recognition as part of the general PSP diagnostic possibilities, the prevalence of MPS has never been characterized in patients with CPSP patients. We performed a cross-sectional standardized clinical and radiological evaluation of patients with definite CPSP in order to assess the presence of other non-neuropathic pain syndromes, and in particular, the role of myofascial pain syndrome in these patients.

**Methods:**

CPSP patients underwent a standardized sensory and motor neurological evaluation, and were classified according to stroke mechanism, neurological deficits, presence and profile of MPS. The Visual Analogic Scale (VAS), McGill Pain Questionnaire (MPQ), and Beck Depression Scale (BDS) were filled out by all participants.

**Results:**

Forty CPSP patients were included. Thirty-six (90.0%) had one single ischemic stroke. Pain presented during the first three months after stroke in 75.0%. Median pain intensity was 10 (5 to 10). There was no difference in pain intensity among the different lesion site groups. Neuropathic pain was continuous-ongoing in 34 (85.0%) patients and intermittent in the remainder. Burning was the most common descriptor (70%). Main aggravating factors were contact to cold (62.5%). Thermo-sensory abnormalities were universal. MPS was diagnosed in 27 (67.5%) patients and was more common in the supratentorial extra-thalamic group (P <0.001). No significant differences were observed among the different stroke location groups and pain questionnaires and scales scores. Importantly, CPSP patients with and without MPS did not differ in pain intensity (VAS), MPQ or BDS scores.

**Conclusions:**

The presence of MPS is not an exception after stroke and may present in association with CPSP as a common comorbid condition. Further studies are necessary to clarify the role of MPS in CPSP.

## Background

Central poststroke pain (CPSP) is a neuropathic pain syndrome associated with somatosensory abnormalities due to central nervous system (CNS) lesion following a cerebrovascular insult. CPSP pain has been reported since the end of the XIX century [1]. Classically described after vascular lesions in the thalamus [2], CPSP is also common in lesions of the dorso-lateral medulla [3], thalamic-capsular [4], and parietal regions [5]. It can be a consequence of lesions located anywhere along the telencephalon involving the somatosensory pathways [6,7]. It has been demonstrated that multiple vascular encephalic lesions are present in the majority of CPSP patients [4].

Poststroke pain (PSP) refers to a broader range of clinical conditions leading to pain after stroke, but not restricted to pain of central neuropathic nature (CPSP). PSP affects from 11 to 55% of patients following a CNS vascular event [8-10]. It includes several painful conditions such as CPSP, painful shoulder, lumbar and dorsal pain, complex regional pain syndrome, tension type headache and spasticity-related pain [10-12]. In particular, musculoskeletal pain is prevalent in PSP patients [13], being probably secondary to decreased muscle strength and altered descending modulatory system tonus [14]. Myofascial pain syndrome (MPS) is defined by the occurrence of regional pain and stiffness, limited range of motion in the affected muscle, satellite trigger points and twitch response to palpation of taut bands in the muscles [15,16].

MPS can also serve as a peripheral pain generator and alter the function of descending modulatory pathways as has been recently reported in other chronic pain conditions [17]. Besides the obvious implications of the presence of musculoskeletal pain in PSP on treatment, it may also pose diagnostic difficulties due to the presence of referred pain that commonly accompanies myofascial pain syndromes. Despite its recognition as part of the general PSP diagnostic possibilities, the prevalence of MPS has never been characterized in patients with CPSP. The identification of the co-occurrence of MPS with CPSP pain is of major importance for two reasons: first, using the current diagnostic criteria of neuropathic pain [18], MPS pain can be erroneously considered as neuropathic pain since it also refers to the presence of pain in an area of somatosensory system lesion. Second, and most important, it has been recently proposed that CPSP should be a diagnosis of exclusion, since there would be no pathognomonic feature of the syndrome [19], being reserved for patients with PSP and without other clear nociceptive or peripheral neuropathic pain syndromes. Although apparently sound, this proposal must be tested, since it is not known to which extent CPSP overlaps with other PSP syndromes such as MPS.

We performed a cross-sectional standardized clinical and radiological evaluation of patients with definite CPSP in order to assess the presence of MPS in this sample.

## Methods

Forty CPSP patients were evaluated in the Pain Center of the Hospital das Clínicas in the University of São Paulo. All patients presented at least one stroke affecting the somatosensory pathways, as documented by brain MRI or CT scans.

Inclusion criteria were adults (> 18 years), presenting definite neuropathic pain with sensory deficits in the same topographic area related to a lesion to the somatosensory system [18]. Exclusion criteria were the presence of pain of exclusive nociceptive or peripheral neuropatic origin, the presence of major aphasia or other cognitive deficit impairing the report of the sensory abnormalities and pain characteristics.

### Clinical evaluation

All patients signed an informed consent to participate in the study. The protocol was approved by our local Ethics Review Board. Patients were asked to keep current medications and were assured to have their treatment continued during and after the end of the study.

Data on associated health conditions, time between stroke and pain onset, and duration of pain were recorded during a structured interview.

Patients were instructed to indicate the site of their neuropathic pain in a human body template and to identify the presence and location of the other painful areas.

**Figure 1:**
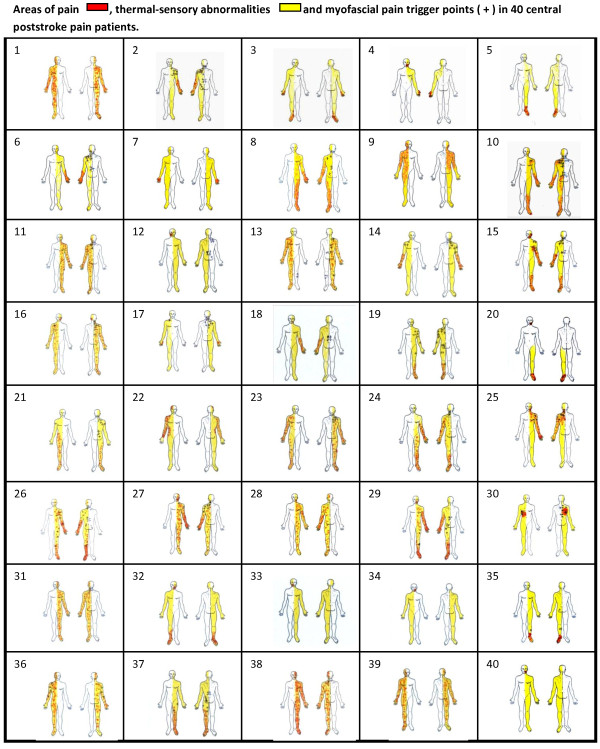
**Pain area, thermal deficits and miofascial pain syndrome trigger points in central poststroke pain patients.** Areas of pain (red), thermo-sensory abnormalities (yellow) and myofascial pain trigger points ( + ) in 40 patients with central poststroke pain.

Bedside physical examination was performed with the following tests: vibration detection threshold was performed with a 128 Hz vibrating tuning fork applied to the first finger and to the toe bilaterally. Thresholds were defined as the time elapsed from the beginning of the exam to the point where the patients ceased to detect the vibration stimulus. Hyperalgesia was assessed with a pinprick. Mechanical dynamic allodynia was assessed with a soft brush slightly stroke for 6 cm 2 cm/sec. Tactile non-painful stimulus was investigated with a cotton swab. Thermal sensitivity was assessed with hot (40-45°C) and cold (5-10°C) water-filled tubes. Thermal allodynia was defined as the presence of pain to the contact of a glass tube containing water at 20°C in the absence of mechanical allodynia. Each sensory test was performed in predetermined cutaneous points five centimeters apart from each other from the face to the feet bilaterally [20,21] (Figure 1). MPS was searched for in a systematic manner by gentle manual palpation of predefined muscles trigger points. When palpation elicited the characteristic regional referred pain from the muscle being tested, trigger points were considered as active and MPS was defined after the other diagnostic criteria were fulfilled [15,16]. The main muscles evaluated for MPS in CPSP patients were defined according to a pilot study previously performed in a similar group of patients. The location of the active trigger points, the extension of sensory deficit and the location of pain was marked and a human body template by the examiner.

Pain intensity was assessed by the visual analogic scale at the moment of the evaluation (VAS) [22]. The different aspects of pain were measured by the McGill Pain Questionnaire (MPQ) [23] adapted to the Portuguese language [24]. Mood was assessed by the Beck depression scale [25].

Brain MRI was performed in 24 patients (65%) or CT scan in 16 (35%). Lesions sites were classified as thalamic, thalamic-capsular, supratentorial extra-thalamic, brain stem or undetermined by a blinded experienced neuroradiologist. Patients with multiple encephalic lesions were stratified according to main site of the lesion anatomically related to the sensory deficits.

Patients were on regular pharmacotherapy for pain control when data were collected. They used, as mono or polytherapy, the following medications: heterocyclics antidepressants in 33 (82.5%) patients and anticonvulsants in 21 (52.5%) (Table 1). Regular physical therapy was performed in 5 (12.5%) and acupuncture in 6 (15.0%).

**Table 1:** Patient’s demographics, types of stroke, past medical history, VAS, aggravating factors of pain, the presence of motor deficits, MPS and medications in 40 patients with central poststroke pain

**Patient**	**Age**	**Gender**	**Stroke**	**Past medical hystory**	**VAS**	**Aggravating factors**	**Motor deficits**^**1**^	**MPS**	**Medications**	**One year follow-up VAS**
**1**	**65**	**male**	**Ischemic**	**Hipertension**	**10**	**cold / movement / mood swings**	**no**	**no**	**nortrip, CBZ, methad**	**7**
**2**	**53**	**female**	**Ischemic**	**Diabetis**	**10**	**cold / mood swings**	**yes**	**yes**	**amitrip, CBZ, NSAID**	**8**
**3**	**60**	**male**	**Ischemic**	**Hypertension**	**8**	**cold / movements**	**no**	**no**	**amitrip, GBP**	**2**
**4**	**65**	**female**	**Ischemic**		**10**	**weather changes / cold**	**no**	**no**	**nortrip, CBZ**	**8**
**5**	**69**	**male**	**Ischemic**		**6**	**mood swings**	**yes**	**no**	**amitrip, CBZ, chlorpr**	**0**
**6**	**49**	**male**	**Ischemic**	**Hypertension**	**10**	**heat / mood swings**	**yes**	**yes**	**GBP**	**8**
**7**	**63**	**female**	**Ischemic**	**Hypertension**	**9**	**cold /movements**	**no**	**no**	**amitrip**	**4**
**8**	**54**	**male**	**Ischemic**	**Diabetis**	**10**	**mood swings**	**no**	**yes**	**amitrip, CBZ, GBP, tramadol**	**10**
**9**	**64**	**male**	**Ischemic**		**10**	**cold / movements**	**yes**	**no**	**imipramine, chlorpr**	**8**
**10**	**45**	**female**	**Ischemic**	**Hypertension**	**10**	**cold / mood swings**	**no**	**yes**	**amitrip, sertraline, CBZ, chlorpr**	**6**
**11**	**74**	**male**	**hemorragic**		**8**	**cold / weather changes / mood swings**	**yes**	**yes**	**amitrip**	**4**
**12**	**61**	**male**	**Ischemic**	**Hypertension**	**10**	**cold / weather changes /movements**	**yes**	**yes**	**CBZ, cyclobenz**	**3**
**13**	**53**	**female**	**Ischemic**	**Cardiopathy, diabetis**	**10**	**diabetis descompensation**	**yes**	**yes**	**amitript**	**8**
**14**	**65**	**male**	**Ischemic**	**Hypertension**	**9**	**cold / heat / mood swings**	**no**	**yes**	**nortrip**	**7**
**15**	**65**	**male**	**Ischemic**	**Hypertension**	**10**	**mood swings**	**yes**	**yes**	**amitrip, chlorpr**	**5**
**16**	**65**	**male**	**Ischemic**		**10**	**cold / movements / mood swings**	**no**	**yes**	**amitrip, methad**	**6**
**17**	**34**	**female**	**Ischemic**		**10**	**skin contact / movements**	**no**	**yes**	**nortrip, chlorpr**	**8**
**18**	**67**	**male**	**Ischemic**	**Hypertension**	**9**	**cold / contact / movements**	**no**	**yes**	**GBP**	**6**
**19**	**61**	**male**	**Ischemic**	**Cardiopathy**	**9**	**cold / mood swings**	**yes**	**yes**	**nortrip, fluox, baclof, CBZ**	**3**
**20**	**66**	**male**	**Ischemic**	**Hypertension**	**10**	**heat / cold**	**yes**	**no**	**nortrip**	**5**
**21**	**59**	**female**	**Ischemic**	**Parkinsons**	**10**	**mood swings**	**no**	**yes**	**amitrip, chlorpr**	**6**
**22**	**40**	**male**	**Ischemic**	**Hypertension**	**10**	**cold / heat**	**no**	**no**	**amitrip, GBP**	**9**
**23**	**41**	**male**	**hemorragic**	**Hypertension**	**10**	**cold / mood swings**	**yes**	**yes**	**amitrip, CBZ, chlorp**	**10**
**24**	**78**	**female**	**Ischemic**	**Hypertension**	**5**	**cold / mood swings**	**yes**	**yes**	**imipramin, chlorp**	**4**
**25**	**80**	**male**	**Ischemic**	**Diabetis**	**6**	**diabetis descompensation / heat**	**yes**	**yes**	**amitrip, CBZ, chlorp**	**4**
**26**	**84**	**female**	**Ischemic**	**Hypertension**	**10**	**mood swings**	**no**	**yes**	**amitrip, sertral, bromazep**	**9**
**27**	**55**	**female**	**Ischemic**	**Hypertension**	**8**	**cold / movements**	**no**	**yes**	**amitrip, chlorpr**	**5**
**28**	**63**	**female**	**Ischemic**		**8**	**mood swings / movements**	**no**	**yes**	**amitrip, sertral**	**5**
**29**	**60**	**male**	**Ischemic**		**8**	**sexual activity / movements**	**no**	**yes**	**clomipramine, CBZ**	**8**
**30**	**56**	**male**	**Ischemic**	**Hypertension**	**8**	**cold / heat**	**no**	**yes**	**amitrip, chlorpr**	**6**
**31**	**64**	**male**	**Ischemic**		**7**	**cold / weather changes**	**no**	**no**	**GBP**	**6**
**32**	**60**	**male**	**Ischemic**	**Migraine**	**6**	**cold / movement / mood swings**	**no**	**no**	**CBZ**	**0**
**33**	**47**	**male**	**Ischemic**		**10**	**cold / weather changes**	**yes**	**yes**	**amitrip, GBP**	**8**
**34**	**64**	**male**	**Ischemic**	**Hypertension**	**7**	**mood swings / movements**	**no**	**no**	**amitrip, CBZ**	**0**
**35**	**51**	**male**	**Ischemic**	**Diabetis**	**9**	**cold / heat**	**yes**	**yes**	**amitrip, cyclobenz**	**7**
**36**	**42**	**female**	**hemorragic**		**9**	**mood swings**	**no**	**yes**	**amitript**	**8**
**37**	**61**	**female**	**Ischemic**	**Hypertension**	**10**	**cold / movement**	**yes**	**yes**	**GBP, nortrip**	**7**
**38**	**54**	**male**	**Ischemic**		**10**	**mood swings / movements**	**yes**	**yes**	**GBP**	**8**
**39**	**67**	**female**	**hemorragic**	**dislipidemia**	**10**	**cold / heat**	**no**	**no**	**amitript**	**9**
**40**	**58**	**male**	**Ischemic**	**Hypertension**	**7**	**mood swings**	**no**	**no**	**amitript**	**5**

MPS = miofascial pain syndrome, 1 = pyramidal motor deficits, amitrypt = amitryptiline, nortrypt = nortriptiline, CBZ = carbamazepine, GBP = gabapentin, chlorpr = chlorpromazine, fluox = fluoxetine, sertral = sertraline, methad = methadone.

### Statistical analyses

Results are expressed as mean (± standard deviation) or median (range) according to the distribution of the variables. Qualitative variables were analyzed according to Fisher’s exact test. The χ^2^ test was used to compare the proportions of patients with pyramidal signs and MPS. Quantitative variables were analyzed by the Kruskall-Wallis test, according to the normal distribution of the data as assessed by the Shapiro-Wilk normality test. For all tests significance was set at P < 0.05.

## Results and discussion

Forty patients were evaluated (26 males, 59.5±10.7 years old, ranging from 34 to 84 years old). Thirty-six (90.0%) had ischemic stroke (s). Thirty (75.0%) patients had one single and six (15%) had more than one ischemic vascular event. Three (7.5%) patients presented intra-cerebral hemorrhage and one (2.5%) subarachnoid hemorrhage followed by vasospasm in the territory of the left middle cerebral artery. Thirty-four (85.0%) patients had systemic arterial hypertension, 7 (17.7%) diabetis mellitus, 5 (12.5%) cardiopathy, 2 (5.0%) hypercholesterolemia, 1 (2.5%) migraine. Major neurologic deficits were motor pyramidal motor syndrome in 17 (42.5%), cranial nerves deficits in 6 (15.0%) cerebellar syndrome in 5 (12.5%), choreoatetosic movements in 3 (7.5%), sensory ataxia in two (5.0%) and hemianopsia in one (2.5%) patient.

Pain onset was insidious in 31 (77.5%) patients and presented during the first three months after stroke in 30 (75.0%) (Table 1). Pain was reported in the whole hemi-body in 22 (55.0%) patients, and had a multifocal distribution in the remainder.

Median pain intensity according to VAS was 10 (5 to 10) and the average pain duration period was 5.73 (±4.39) years. There was no difference in pain intensity among the different lesion site groups. Neuropathic pain was continuous-ongoing in 34 (85.0%) patients and intermittent in the remainder. Burning was the most common descriptor (70%), followed by electric shock-like paroxysms (22.5%). Nineteen (47.5%) patients had more than one descriptor for their neuropathic pain. Main aggravating factors were contact to cold (62.5%), mood swings (52.5%), movement of the painful limb (37.5%), and contact to heat (20%). There was more than one aggravating factor in 33 (82.5%) patients (Table 1). No statistically significant difference was found regarding the descriptors of pain, aggravating factors, and the MPQ scores among the lesion groups. Thermo-sensory abnormalities were universal in the series and are expressed on Table 2.

**Table 2:** Sensory abnormalities according to the location of encephalic lesions in CPSP patients

**Sensory changes**	**Lesion site**					
	**Th**	**ThC**	**SETh**	**BrS**	**Und**	**Total**
Heat and cold hypoesthesia	6 (75.0)	5 (100.0)	14 (100.0)	7 (87.5)	5 (100.0)	37 (92.5)
Heat and cold hyperesthesia	1 (12.5)		-	-	-	1 (2.5)
Cold hyperesthesia and heat hypoesthesia	1 (12.5)	-	-	1 (12.5)	-	2 (5.0)
Hypalgesia	5 (62.5)	4 (80.0)	12 (85.7)	6 (75.0)	3 (60.0)	30 (75.0)
Hyperalgesia	3 (37.5)	1 (20.0)	2 (14.3)	1 (12.5)	2 (40.0)	9 (22.5)
Hyperpathia	6 (75.0)	4 (80.0)	11 (78.6)	5 (62.5)	3 (60.0)	29 (72.5)
Reduced vibration sensitivity	6 (75.0)	3 (60.0)	14 (100.0)	2 (25.0)	3 (60.0)	28 (70.0)
Tactile allodynia	3 (50.0)	3 (60.0)	10 (71.4)	4 (50.0)	2 (40.0)	22 (57.9)
Thermal allodynia	1 (12.5)	4 (80.0)	7 (50.0)	3 (37.5)	3 (60.0)	18 (45.0)
Kinestesic allodynia	-	-	3 (21.4)	-	-	3 (7.5)
TOTAL	8	5	14	8	5	40 (100)

Results are expressed as number (%). Th: Thalamic; ThC: Thalamic-capsular; SETh: Supratentorial extra-thalamic; BrS: Brain Stem; Und: Undetermined. CPSP: Central Post-Stroke Pain.

Painful shoulder syndrome was diagnosed in 4 (10.0%) patients and shoulder-hand syndrome in 1(2.5%). MPS was diagnosed in 27 (67.5%) patients and was more common in the supratentorial extra-thalamic group (P <0.001) (Table 3). It was more frequent in patients with pyramidal deficits (82.35%) than those without it (56.52%), however this difference was not statistically significant (P = 0.017). The main muscles affected by MPS in CPSP patients were presented on Table 4. The spatial relationship between thermal deficits, pain and MPS pain area for each patient is illustrated in Figure 1. Autonomic abnormalities were found in 23 (57.5%) cases: Horner’s sign was observed in 6 (15.0%) cases, hypothermia of the upper and lower extremities in 10 (25.0%) patients, hyperemia in 9 (22.5%), edema in 5 (12.5%), hyperhydrosis in 2 (5.0%) and pallor in 2 (5.0%). The mean score of the BDQ was 22.87±11.96. No significant differences were observed among the different stroke location groups and pain quastionaires and scales scores. Importantly, CPSP patients with and without MPS did not differ in pain intensity (VAS), MPQ or BDS scores.

**Table 3:** Presence of Myofascial Pain Syndromes according to stroke location

	**MPS present**	**MPS absent**	**Total**	**p**
Th	5 (50%)	5 (50%)	10 (25%)	n.s.
ThC	0 (0%)	5 (100%)	5 (12.5%)	n.s.
SETh	1 (7.1%)	13 (92.9%)	14 (35%)	P <0.001
BrS	5 (62.5%)	3 (37.5%)	8 (20%)	n.s.
Und	3 (60%)	2 (40%)	5 (12.5%)	n.s.
Total	13 (32.5%)	27 (67.5%)	40 (100%)	n.s.

Results are expressed as number (%). Th: Thalamic; ThC: Thalamic-capsular; SETh: Supratentorial extra-thalamic; BrS: Brain Stem; Und: Undetermined.

**Table 4:** Muscles affected by Myofascial Pain Syndrome in Central Post Stroke Pain Patients

**Muscle**	**Number of patients (%)**
**Scalenus**	**3 (7,5)**
**Sternocleidomastoideus**	**1 (2,5)**
**Splenius**	**12 (30)**
**Semispinalis**	**11 (27,5)**
**Trapezius**	**20 (50)**
**Levator scapulae**	**10 (25)**
**Supraspinatus**	**12 (30)**
**Infraspinatus**	**8 (20)**
**Pectoralis**	**8 (20)**
**Rhomboideus**	**10 (25)**
**Latissimus dorsi**	**1 (2,5)**
**Paravertebral dorsi**	**1 (2,5)**
**Paraverterbal lomborum**	**12 (30)**
**Quadratus lomborum**	**8 (20)**
**Deltoid**	**9 (17,5)**
**Biceps brachii**	**3 (7,5)**
**Triceps brachii**	**6 (15)**
**Brachioradialis**	**3 (7,5)**
**Glutaeus**	**6 (15)**
**Piriformis**	**7 (17,5)**
**Tractus ileospinalis / tensor fascia latae**	**5 (12,5)**
**Triceps surae**	**5 (12,5)**
**Tibialis anterior**	**3 (7,5)**

We performed a clinical-radiological evaluation of a group of CPSP patients focusing on the co-occurrence of PSP syndromes other than CPSP, in particular the role of MPS in these patients. CPSP was diagnosed according to the definite revised neuropathic pain criteria [18]. The presence of thermo-sensory deficits in all patients is a clinical hallmark of central pain, described in other series [4,26-28]. We looked for the presence MPS in a standardized fashion. MPS was present in the majority of cases (67.5%), suggesting that “pure” neuropathic pain syndrome is present in the minority of CPSP. Instead, most patients had mixed pain syndromes in which central neuropathic pain was associated with other syndromes (nociceptive). It has been recently proposed that CPSP should be a diagnosis of exclusion in PSP patients [19]. Our data suggest that this proposal might lead to under-diagnose of CPSP in PSP patients with associated MPS. A clear limitation of the design of our study is the lack of a control group. Evaluating the presence of MPS in a PSP population without CPSP would help us to better understand the factors influencing the occurrence of MPS in PSP patients. However, even if PSP patients without CPSP had a high prevalence of MPS, the prevalence of MPS in CPSP would still be quite high (as shown here), suggesting that for nomenclature and definition purposes CPSP should not be a diagnosis of exclusion. Certainly, this assumption must be confirmed by other larger studies, as well as the role of MPS in pain treatment and rehabilitation in these individuals. Furthermore, the identification of a central neuropathic element in a pain of musculoskeletal origin can be difficult and in some cases, several pain types might be present in the same area of the body [19], the findings of our study corroborate to this view. Motor deficits, spasticity, movements disorders and altered central descending pain modulation [14,29] may all induce overload to the muscles and trigger myofascial pain [2,4,5,8,10-12,27]. MPS in its turn can serve as peripheral generator of nociceptive inputs that may alter pain perception, as has been suggested to occur in other chronic pain conditions [17]. MPS was more frequent in supratentorial extra-thalamic (92.9%) and thalamic-capsular (100%) subgroups when compared with thalamic (50.0%) and brain stem (37.5%) stroke groups. This finding could be a consequence of the magnitude of the motor deficits and spasticity; however, we found no association on the presence of pyramidal deficits and the presence of MPS. Many patients in the supratentorial extra-thalamic group presented extensive brain lesions, usually secondary to occlusion of major arteries, resulting in major motor deficits, postural abnormalities, hypertonia and spasticity. This is further supported by the finding that 4 of the 8 patients in the thalamic group had an infarction located in the territory of the thalamic-geniculate artery, encompassing most of the sensory pathways while preserving motor cortical-spinal fibers [30,31].

To our best knowledge, the prevalence of MPS in patients with CPSP has not been described previously. Interestingly, the presence of MPS was not associated with more intense pain or pain associated mood disorders in CPSP patients, however, this can be due to some limitations of the study, such a small number of patients and a ceiling effect related to the high pain intensity of these highly refractory individuals. Also, we did not quantify the intensity of MPS, which could help better understand its relationship to pain intensity and disability. In our sample of CPSP individuals the mean pain duration period was high (5.73 years), the pain scores, as well as the depression rates, were elevated, indicating a condition of high chronicity, psychosocial stress and refractoriness to treatment. The limited access of the stroke patients to a physical therapy program, performed in only five (12.5%) patients could have increased the incidence of MPS in this series. Moreover, a comparative analysis of the CPSP series with a control group was not performed. Still, our data suggest that MPS should be viewed as a common comorbid condition co-occurring with the CPSP syndrome complex. Similarly, we found painful shoulder in four (10.0%) patients and shoulder-hand syndrome in one (2.5%). Shoulder-hand syndrome is caused by glenohumeral joint subluxation due to motor paresis and is commonly associated with painful shoulder and is one of the possible presentations of the complex regional pain syndrome in PSP patients [12,32,33]. Autonomic abnormalities were found in 57.5% of our patients. Many CPSP patients present motor deficits and avoid movement of painful parts of the body. Prolonged immobilization may induce sensory, neurovegetative, motor and trophic abnormalities that can worsen pain and induce complex regional pain syndrome, a condition associated with neurovegetative abnormalities. [33,34] Autonomic dysfunction can also be related to the encephalic lesion such as lateral medullary stroke and Wallenbergs Syndrome in 4 (10.0%) patients.

Neurological deficits add more suffering to that already caused by pain and psychosocial problems related to handicap. Chronic suffering and incapacitation often lead to, or facilitate the onset of depression. There is a close relationship between pain and depression [35,36] and the occurrence of depressive states in stroke patients is a well-known phenomenon [10,37]. Leijon et al. [10] reported higher incidence of depression in CPSP patients than in a control group. Andersen et al. [28] found no positive correlations for depression when stroke patients with somatosensory deficits with and without CPSP were compared. In our series, the mean score of the BDQ was 22.87 and the prevalence of moderate and severe depressive states were high. Recognizing and treating this condition is equally important if one takes into consideration that central pain can further increase the negative impact of depression on quality of life and increase the suicide risk [38].

## Conclusions

We diagnosed MPS in more than two thirds of CPSP patients. It was more common in patients with lobar and thalamic-capsular lesions although no association was found between motor deficits and a higher prevalence of MPS. The presence of MPS is not an exception in CPSP and may represent a common comorbid condition. The impact of MPS in pain treatment, prognosis and its role in rehabilitation of these patients remain to be determined. Our results should provide insights on the current diagnostic criteria of CPSP and draw attention to the different pain syndromes in the PSP syndrome complex.

## Competing interest

The author(s) declare that they have no competing interests.

## Authors contributions

RAAO participated in study design, data collection, manuscript writing and review; DCA participated in statistical analyses and manuscript review; AGGM carried out data entry and analyses; MJT participated in study design and manuscript review. All authors read and approved the final manuscript.

## Pre-publication history

The pre-publication history for this paper can be accessed here:

http://www.biomedcentral.com/1471-2377/12/89/prepub
